# Prediction of tumour sensitivity to 4-hydroperoxycyclophosphamide by a glutathione-targeted assay.

**DOI:** 10.1038/bjc.1991.52

**Published:** 1991-02

**Authors:** F. Y. Lee, D. J. Flannery, D. W. Siemann

**Affiliations:** Department of Radiation Oncology, University of Rochester Cancer Center, New York 14642.

## Abstract

In an attempt to develop an assay to predict patient tumour response to cyclophosphamide (CP), the feasibility of using a glutathione-targeted assay to assess the in vitro chemosensitivity of tumour cells to 4-hydroperoxycyclophosphamide (4-OOH-CP), an activated congener of CP, was evaluated. A panel of 19 human and three murine tumour cell lines was used. These consisted of three main categories of tumour types, viz. ovarian, lung and squamous cell carcinoma. The major finding was that the occurrence of a significant reduction of tumour cell reproductive capacity was always accompanied by substantial depletion of cellular glutathione (GSH) content, and vice versa. Plots of % GSH depletion versus clonogenic cell survival demonstrated highly significant correlation (r = 0.90-0.91; P less than 0.01). It was determined that for in vitro tumour cell lines, a GSH depletion to 40% of initial content may serve as a cut-off criterion for chemosensitivity to 4-OOH-CP. This degree of GSH depletion is indicative of clonogenic cell survival of approximately 1% (95% confidence limits = 3 x 10(-5)-1.6 x 10(-2)). The relationship between steady state GSH content and intrinsic sensitivity to 4-OOH-CP was also evaluated. The GSH concentration of the tumour cell lines ranged from 1.3-21.2 x 10(-18) moles microns-3; chemosensitivity to 4-OOH-CP, in terms of IC99, was in the range of 5.0-87.1 microM. A good correlation was observed between these two parameters (r = 0.85, P less than 0.02). These results suggest that GSH plays an important role in determining the therapeutic efficacy of 4-OOH-CP in the treatment of cancer. It is uncertain, however, whether a high tumour steady state GSH content in itself is sufficient to cause therapeutic failure in patients.


*Br. J. Cancer* (1991), **63**, 217–222 © Macmillan Press Ltd., 1991

### Prediction of tumour sensitivity to 4-hydroperoxycyclophosphamide by a glutathione-targeted assay*

F.Y.F. Lee, D.J. Flannery & D.W. Siemann

*Experimental Therapeutics Division and Department of Radiation Oncology, University of Rochester Cancer Center, Rochester, New York 14642, USA.*

**Summary** In an attempt to develop an assay to predict patient tumour response to cyclophosphamide (CP), the feasibility of using a glutathione-targeted assay to assess the *in vitro* chemosensitivity of tumour cells to 4-hydroperoxylcyclophosphamide (4-OOH-CP), an activated congener of CP, was evaluated. A panel of 19 human and three murine tumour cell lines was used. These consisted of three main categories of tumour types, viz. ovarian, lung and squamous cell carcinoma. The major finding was that the occurrence of a significant reduction of tumour cell reproductive capacity was always accompanied by substantial depletion of cellular glutathione (GSH) content, and vice versa. Plots of % GSH depletion versus clonogenic cell survival demonstrated highly significant correlation ($r = 0.90-0.91$; $P < 0.01$). It was determined that for *in vitro* tumour cell lines, a GSH depletion to 40% of initial content may serve as a cut-off criterion for chemosensitivity to 4-OOH-CP. This degree of GSH depletion is indicative of clonogenic cell survival of approximately 1% (95% confidence limits = $3 \times 10^{-5}-1.6 \times 10^{-2}$). The relationship between steady state GSH content and intrinsic sensitivity to 4-OOH-CP was also evaluated. The GSH concentration of the tumour cell lines ranged from $1.3-21.2 \times 10^{-18}$ moles $\mu m^{-3}$; chemosensitivity to 4-OOH-CP, in terms of $IC_{99}$, was in the range of 5.0–87.1 μM. A good correlation was observed between these two parameters ($r = 0.85$, $P < 0.02$). These results suggest that GSH plays an important role in determining the therapeutic efficacy of 4-OOH-CP in the treatment of cancer. It is uncertain, however, whether a high tumour steady state GSH content in itself is sufficient to cause therapeutic failure in patients.

Cyclophosphamide (CP) and its congeners are among the most efficacious and frequently used agents for the treatment of human malignancies. The pharmacology and metabolic fate of CP has been extensively studied and is now relatively well understood (Sladek, 1987). When given systemically, CP is metabolised, through hydroxylation by the hepatic mixed-function oxidases, to 4-hydroperoxycyclophosphamide (4-OH-CP), a 'transport' form of CP which exists in equilibrium with its ring-opened tautomer aldophosphamide (AP). Glutathione (GSH) can participate in conjugative reactions at two separate locations in the cyclophosphamide metabolic pathway that may exert considerable influence on the eventual cytotoxicity of the cytotoxic agent (see Figure 1 of the accompanying paper (Lee, 1990) and others (Draeger *et al.*, 1976; Gurtoo *et al.*, 1981)). (1) GSH reacts with AP to form 4-OH-CP and shifts the tautomerism pseudo-equilibrium between 4-OH-CP and aldophosphamide (AP) in favour of the former tautomer (Draeger *et al.*, 1976; Lee, 1990). (2) GSH binds irreversibly to the toxic metabolites of 4-OH-CP, in particular acrolein (Gurtoo *et al.*, 1981). Because of these reactions, exposure of cells to 4-OH-CP may lead to depletion of endogenous GSH if the combined rates of the conjugation reactions exceed the rate of GSH recovery. In fact, a number of studies, all using high and therefore toxic doses of CP, have already shown that CP depletes cellular GSH level *in vivo* (Adams *et al.*, 1986; Carmichael *et al.*, 1986) and *in vitro* (Crook *et al.*, 1986). Recently, in a series of experiments in which both GSH status and cytotoxicity were determined in the same tumour cell population, we observed that toxic concentrations of 4-OOH-CP† severely depleted tumour cells of GSH whereas nontoxic concentrations of 4-OOH-CP produced no such effect (Siemann *et al.* in preparation). Furthermore, a tumour cell type (KHT) highly susceptible to GSH depletion by 4-OOH-CP was also found to be highly sensitive to this agent while a tumour cell line (A549) resistant to GSH depletion by 4-OOH-CP was shown also to be refractory to the drug's cytotoxic effects. The present study was therefore designed to: (1) establish in detail the relationship between 4-OOH-CP induced GSH depletion and cytotoxicity and (2) determine whether measurements of the extent of GSH depletion induced by clinically relevant concentrations of 4-OOH-CP can be used to predict for tumour response to this anticancer agent.

### Materials and methods

### *Cell culturing*

Three major categories of human tumour cell lines were used in this study: ovarian, lung and squamous cell carcinomas. The ovarian cell lines, except SKOV-3, CaOV-3 and NIH: OVCAR-3, were established from biopsy specimens (Lee *et al.*, 1989). SKOV-3, CaOV-3 and NIH:OVCAR-3 were obtained from the American Type Culture Collection. The small cell lung line POC was received from Dr P. Twentyman. The other lung lines were obtained from Dr J. Mitchell courtesy of Dr J. Minna. Three epidermoid cell lines were also used in this study. They were HEp3 derived from a metastatic buccal mucosa carcinoma (Toolan, 1954), A431 derived from a vulva carcinoma (Giard *et al.*, 1973), and ME180 derived from a cervix carcinoma (Sykes *et al.*, 1970). Limited studies with the rodent KHT sarcoma cell line (Kallman *et al.*, 1967) were performed. Culture conditions, growth characteristics and plating efficiency for the human tumour cell lines are listed in Table I. All nutrient media and sera were obtained from Gibco Laboratories (Grand Island, NY). Cell cultures were maintained at 37°C under an atmosphere of 5% $CO_2$/95% air. All cell lines, except POC, grew as monolayers and were routinely subcultured every 3 to 4 days with standard trypsinisation procedures using Worthington's trypsin (Worthington Biochemical Corp, New Jersey, USA). Clonogenic assays were performed with exponentially growing cells 2 to 3 days after seeding.

*Supported by PHS Grant CA-38637.
Correspondence: F.Y.F. Lee.
Received 8 March 1990; and in revised form 7 September 1990.

†4-OOH-CP gives rise rapidly to 4-OH-CP following dissolution without any enzymic involvement and may be regarded as equivalent to 4-OH-CP pharmacologically (Sladek, 1987). 4-OOH-CP is the preferred 'activated' cyclophosphamide for routine use only because of its higher stability in crystalline state and easier synthesis.

**Figure 1** Clonogenic cell survival of three main categories of human tumour cell lines following treatment with various concentrations of 4-OOH-CP for 3 h. Each datum point is the mean surviving fraction determined from the colony counts of 10–15 plates. Standard errors are for all instances <10% of the mean. Error bars when shown, are ±1 s.d. Results are from at least three repeat experiments.

**Table I** Characteristics and treatment histories of the human tumour cell lines investigated

| *Cell lines* | *Tumour types* | *Medium* | *FCS (%)* | *Ploidy Ratio* | $T_D$ *(h)* | *CFE (%)* | *Prior treatment* | *Mean cell volume (μm³)* |
|---|---|---|---|---|---|---|---|---|
| *Human ovarian* | | | | | | | | |
| ATW | OVAC | α-MEM | 10 | 2.4 | 48 | 9 | C,A,P | 5,600 |
| PEA | OVAC | α-MEM | 10 | 1.4 | 72 | 14 | C,A,P | 2,700 |
| GRA | OVAC | α-MEM | 10 | 1.5 | 96 | 25 | None | 3,400 |
| MLS | OVAC | α-MEM | 10 | 1.8 | 30 | 72 | C,A,P,F,Mx,Tf | 2,800 |
| SKA | OVAC | α-MEM | 10 | 1.8 | 48 | 37 | C,A,P.F,Mx,Tf | 4,500 |
| SAU | OVAC | α-MEM | 10 | 1.8 | 58 | 38 | None | 8,500 |
| OW-1 | OVAC | α-MEM | 10 | 2.1 | 24 | 85 | C,A,P,M,Mx | 2,500 |
| SKOV-3 | OVAC | α-MEM | 10 | 2.2 | 48 | 26 | T | 2,700 |
| CaOV-3 | OVAC | α-MEM | 10 | 2.0 | 48 | 6 | C,A,F | 5,000 |
| NIH:OVCAR-3 | OVAC | α-MEM | 10 | 1.9 | 88 | 15 | C,A,P | 5,400 |
| *Human lung* | | | | | | | | |
| A549 | LAC | RPMI-1640 | 10 | – | 24 | 60 | N.A. | 2,500 |
| NCI-H520 | LSqC | RPMI-1640 | 10 | – | 24 | 75 | None | 2,600 |
| NCI-H226 | LSqC | RPMI-1640 | 10 | – | 24 | 15 | None | 4,550 |
| NCI-H157 | LCLC | RPMI-1640 | 10 | – | 24 | 55 | None | 3,300 |
| POC | SCLC | RPMI-1640 | 10 | | 96 | 10 | N.A. | 970 |
| *Human epidermoid* | | | | | | | | |
| A431 | EpCa | D-MEM | 10 | 1.9 | 26 | 55 | None | 3,000 |
| HEp3 | EpCa | F-12 | 20 | 1.9 | 27 | 40 | None | 4,400 |
| ME180 | EpCa | F-12 | 10 | 1.5 | 28 | 70 | Rx | 1,900 |
| *Human breast* | | | | | | | | |
| SKBR3 | BCa | RPMI-1640 | 10 | – | 48 | 12 | N.A. | 2,900 |
| H50578 | BCa | RPMI-1640 | 10 | – | 54 | 15 | N.A. | 3,100 |
| *Rodent* | | | | | | | | |
| KHT | MFSa | α-MEM | 10 | 1.9 | 20 | 55 | None | 1,800 |
| CHO/AUXB1 | CHO | F-10 | 10 | – | 12 | 100 | None | 1,300 |
| CHCR[5] | CHO | F-10 | 10 | – | 18 | 100 | Colchicine | 1,600 |

OVAC, ovarian adenocarcinoma; LAC, lung adenocarcinoma; LSqC, lung squamous carcinoma; LCLC, large cell lung cancer; SCLC, small cell lung cancer; EpC, epidermoid squamous carcinoma; BCa, breast carcinoma; MFSa, murine fibrosarcoma; CHO, Chinese hamster ovary; FCS, foetal calf serum; TD, population doubling time; CFE, colony-forming efficiency. Therapy received before establishment of cell line: c, cyclophosphamide; A, adriamycin; P, cisplatin; F, 5-fluorouracil; M, melphalan; Mx, methotrexate; Tf, tamoxifen; T, thiotepa; N.A., information not available.

*Drug preparation and treatment*

For drug treatment, cells were trypsinised and a single-cell suspension at $2.5 \times 10^5$ cells $ml^{-1}$ in complete medium was placed in a type I vial at 37°C and continuously gassed with a 5% $CO_2$/95% air gas mixture as previously described (Whillans & Rauth, 1980; Siemann *et al.*, 1989). Cells were allowed to equilibrate at these conditions for 30 min prior to drug exposure. Drug treatment was for a 3 h period.

4-hydroperoxycyclophosphamide (4-OOH-CP) and deschloro-4-hydroperoxycyclophosphamide (deschloro-4-OOH-CP) were generous gifts from Dr R.F. Borch (Department of Pharmacology, University of Rochester, USA) and Dr P. Hilgard (ASTA-Werke Bielefeld, FRG). Both agents were dissolved in phosphate-buffered saline (PBS), pH 7.4, and 10 μl of the solution were diluted in 1 ml of culture media, containing the cell suspension being tested, to obtain the final concentration.

*Clonogenic cell survival assay*

At the end of the drug exposure, cells were washed by centrifugation through 14 ml of cold medium for 10 min at 1,000 *g*. The cell pellet was resuspended in complete medium plus foetal calf serum, and the cells were counted with a

Coulter Counter (Model ZBI, Hialeah, FL). Various number of cells were plated in cell culture dishes and 12–18 days later, depending on the cell line, the dishes were stained with crystal violet and survival was determined by counting colonies having more than 50 cells.

### *Glutathione analysis*

Following and at select times during drug exposure, aliquots of cells were removed for HPLC analysis of GSH. Soluble GSH was extracted from cells by homogenisation with 200 µl of 20 mM 5-sulfosalicylic acid. The homogenates were centrifuged for 40 s (Eppendorf Microcentrifuge). GSH was derivatised to a fluorescent conjugate by reacting an aliquot (180 µl) of the supernatant with 12 µl of N-ethylmorpholine (0.5 M in 2.0 mM KOH) and 2 µl monobromobimane (50 mM in acetonitrile, Calbiochem). The reaction mixtures were immediately vortexed and stored in the dark at 4°C until analysis.

Cellular GSH concentrations were measured by pair-ion HPLC as previously described in detail (Lee *et al.*, 1988). Briefly, GSH was separated on Waters Radial-PAK reversed-phase bonded octadecylsilane cartridge columns (C18, 8 mm inner diameter, 5 µm diameter spherical particles). The mobile phase consists of 23% acetonitrile in 40 mM ammonium phosphate buffer, pH 7.2, containing 5 mM tetra-butylammonium hydroxide. The elution condition was isocratic at a flow rate of 3 ml $min^{-1}$ and a total run time of 5 min/sample.

### Results

### *Clonogenic cell survival*

Cell survival curves for the three categories of tumour cell types, following treatments with various concentrations of 4-OOH-CP *in vitro*, are shown in Figure 1. These cell lines had vastly different sensitivities to 4-OOH-CP. The $IC_{50}$, $IC_{90}$ and $IC_{99}$ values ranged from 0.74–25.5, 2.5–56, and 5.0–87.0 µM respectively (Table II). Interestingly, most of the cell lines (four out of five) derived from squamous cell carcinomas were resistant to 4-OOH-CP even though none of them had previously been exposed to chemotherapeutics (Table I). On the other hand the ovarian tumour cell lines, some having been previously exposed to cyclophosphamide, showed a wide spectrum of sensitivities to 4-OOH-CP, ranging from the extremely sensitive (e.g. GRA) to the very resistant (e.g. SKOV-3). These characteristics are very much in accordance with the clinical behaviour of these tumour types to cyclophosphamide treatment.

### *Cellular GSH content at steady-state*

Table II lists the GSH content of all cell lines used. The experiments were internally controlled such that tumour cells used for GSH determinations were drawn from the same pool of cells as those used for chemosensitivity assays. GSH contents varied markedly with tumour cell line, ranging from 1.3 to 21.2 × $10^{-18}$ moles $\mu m^{-3}$. Normalisation of GSH contents with respect to cell volume was essential because the different cell types have diverse cell volumes (Table I), the range being 967–8,500 $\mu m^3$.

### *The relationship between steady-state GSH level and clonogenic cell survival*

Table II and Figure 2 depict the relationship between the sensitivity of the various cell lines to 4-OOH-CP, in terms of $IC_{50}$, $IC_{90}$ and $IC_{99}$, and their steady-state GSH levels. Two definite conclusions can be drawn from these results. (1) There appears to be a direct correlation between the GSH content of a cell type and its resistance to 4-OOH-CP. The goodness of correlation was dependent on the survival end-point chosen as the indicator of sensitivity. The greater the degree of cell kill chosen (e.g. $IC_{90}$ *vis a vis* $IC_{50}$) the better was the correlation. Thus, progressively higher correlation coefficients of 0.78, 0.83 and 0.85 were obtained for steady-state GSH content versus $IC_{50}$, $IC_{90}$ and $IC_{99}$ respectively. (2)

**Table II** Origin, sensitivity and GSH content of cancer cell lines tested using the glutathione-targeted drug-sensitivity assay

| *Cell lines*[a] | *$IC_{50}$ Conc. (µM)* | *$IC_{90}$ Conc. (µM)* | *$IC_{99}$ Conc. (µM)* | *GSH Content (× $10^{-18}$ mol $\mu m^{-3}$)* |
|---|---|---|---|---|
| *Human ovarian ca.* | | | | |
| PEA | 4.0±0.7 | 14.0±2.1 | 28.6±7.2 | 5.5±0.5 |
| GRA | 2.5±0.5 | 8.0±1.5 | 15.6±3.9 | 6.8±0.7 |
| MLS | 7.0±1.6 | 16.3±2.5 | 29.6±10.3 | 12.5±1.0 |
| MLS/$CP^R$ | 13.8±4.1 | 34.5±7.2 | 87.1±23.0 | 16.9±1.8 |
| MLS/$ADR^R$ | 10.4±3.3 | 26.1±6.5 | 58.2±18.7 | 14.3±1.5 |
| SKA | 18.0±3.0 | 29.6±5.3 | 39.0±9.0 | 9.2±0.7 |
| SAU | 15.0±4.8 | 28.0±3.5 | 40.7±11.8 | 8.0±0.9 |
| OW-1 | 3.5±0.8 | 12.5±2.2 | 25.0±5.1 | 6.8±0.4 |
| SK-OV-3 | 20.0±4.4 | 41.5±6.0 | 71.5±15.5 | 18.9±1.4 |
| Caov-3 | 2.3±2.9 | 7.4±1.6 | 15.0±3.2 | 9.0±1.2 |
| NIH:OVCAR-3 | 1.1±0.3 | 4.0±0.5 | 11.0±2.5 | 4.12±0.6 |
| *Human lung ca.* | | | | |
| A549 | 25.0±7.0 | 56.0±6.9 | 84.0±19.0 | 14.4±1.9 |
| NCI-H520 | 20.0±10 | 49.0±5.4 | 78.0±11.6 | 13.2±0.7 |
| NCI-H157 | 1.0±0.2 | 3.5±0.4 | 7.0±1.0 | 4.3±0.5 |
| NCI-H226 | 1.5±0.3 | 4.0±0.7 | 8.0±1.2 | 9.1±0.8 |
| *Human epidermoid* | | | | |
| A431 | 25.5±7.8 | 51.5±9.6 | 78.0±14.4 | 21.3±2.4 |
| HEp3 | 13.0±4.2 | 38.0±6.1 | 73.0±8.1 | 16.1±1.3 |
| ME-180 | 16.0±3.9 | 27.0±8.8 | 46.0±10.9 | 7.8±0.9 |
| *Human breast* | | | | |
| SKBR3 | 3.0±0.7 | 10.6±2.8 | 19.5±5.7 | 7.6±1.1 |
| H50578 | 5.5±1.6 | 15.7±4.4 | 25.6±4.8 | 11.1±2.0 |
| *Rodent lines* | | | | |
| KHT | 0.74±0.2 | 2.50±0.8 | 5.0±0.8 | 1.3±0.2 |
| CHO/AUXB1 | 2.0±0.5 | 6.5±1.6 | 13.0±3.4 | 4.70±0.8 |
| $CHCR^5$ | 4.5±1.2 | 11.6±3.0 | 16.5±5.1 | 6.91±1.3 |

[a]MLS/$CP^R$ and MLS/$ADR^R$ were cell lines selected from the parent MLS line for 4-OOH-CP and ADR resistance by *in vitro* exposure to the respective agents.

**Figure 2** The relationship between steady-state cellular GSH content of 21 tumour cell lines (three murine, 18 human) and chemosensitivity to 4-OOH-CP. Chemosensitivity was expressed in terms of $IC_{50}$, $IC_{90}$ and $IC_{99}$. Error bars represent ± 1 s.d. Each datum point is the average of triplicate determinations.

As shown in Figure 2, there was considerable scattering of the data in the GSH content versus sensitivity plots. Indeed, it is likely that no correlation between GSH content and sensitivity to 4-OOH-CP would have been apparent if only a few cell lines, chosen at random from the 17 reported here, were studied. It therefore appears that steady-state GSH content *per se* cannot serve as an accurate indicator of cellular sensitivity to 4-OOH-CP.

### *The effects of 4-OOH-CP on cellular GSH content*

Incubation of cells with significantly toxic concentrations of 4-OOH-CP invariably led to rapid GSH depletion (compare Figure 3a, b). The rate of depletion was rapid during the initial 60 min, but levelled off considerably thereafter. Recovery of GSH content did not occur during the entire 3 h incubation period. Similar GSH depletion kinetics have previously been reported for the KHT murine fibrosarcoma (Siemman *et al.*, 1989). It should be noted that the loss of cellular GSH following 4-OOH-CP treatment was not caused by leakage to the medium as a result of a loss of membrane integrity. The plasma membrane remained intact following treatment with concentrations of 4-OOH-CP that caused severe reduction of clonogenic capacity. As determined by trypan blue exclusion technique over 95% of cells in all treatment groups retained the ability to exclude the vital dye. In addition, deschloro-4-hydroperoxycyclophosphamide, a vastly less cytotoxic analogue of 4-OOH-CP has been shown to cause severe GSH depletion at non-toxic concentration (Lee, 1990). These results are consistent with the accepted view that the cytotoxic action of 4-OOH-CP resides in its ability to produce DNA crosslinks (Hilton, 1984). On the other hand, acrolien, a breakdown product of 4-OOH-CP released intracellularly is believed to be responsible for the GSH depleting action (Gurtoo *et al.*, 1981; Lee, 1990).

### *The relationship between GSH depletion and cellular sensitivity to 4-OOH-CP*

It was initially observed that when cells were treated with 4-OOH-CP, significant cytotoxicity was always accompanied by significant depletion of GSH content, and vice versa. This observation led us to question whether or not the degree of GSH depletion could be used as a convenient predictor of cellular sensitivity to 4-OOH-CP. A series of internally controlled experiments were performed in which both clonogenic cell survival and GSH depletion were measured on pooled cell populations treated with various concentrations of 4-OOH-CP. Figure 4 summarises the correlative data between cell survival and GSH depletion for the ovarian cancer cell lines (panel a) and the lung and squamous carcinoma cell lines (panel b). Excellent correlations exist between the degree of GSH depletion and cellular sensitivities to 4-OOH-CP. The feasibility of using GSH depletion as a predictor for chemosensitivity is illustrated by the following. At GSH depletion level of 40% of initial content, the toxicity level was ~1% cell survival (95% confidence limits = $3 \times 10^{-5} - 1.6 \times 10^{-2}$). This degree of depletion can therefore be used as the 'cut-off' criterion for sensitivity since cells that were depleted to 40% of their GSH content, or lower, invariably suffered substantial cell kill. Thus cell lines that demonstrated GSH depletion to or beyond the 'cut-off' 40% point with clinically achievable equivalent concentrations of 4-OOH-CP may be considered as sensitive, and vice versa. In addition, a precise definition of sensitivity can be obtained using different concentrations of 4-OOH-CP, as illustrated by Figure 5. The 4-OOH-CP concentration-survival curves of three human tumour cell lines are depicted in panel a. These cell lines have vastly different sensitivities to 4-OOH-CP. This diverse range of sensitivity can be accurately predicted by the GSH assay (panel b). The concentration of 4-OOH-CP needed to produce GSH depletion to ~40% (Figure 5b) of initial level for each cell line was closely related to the corresponding $IC_{99}$ value (Figure 5a and Table II).

### Discussion

The present study demonstrated that an assay technique based on the measurement of the degree of cellular GSH depletion immediately following 4-OOH-CP treatment can effectively and rapidly predict tumour response to 4-OOH-CP. Significant drug cytotoxicity was always accompanied by significant depletion of GSH content and vice versa (Figure 4). For example, a dose of 4-OOH-CP that depletes GSH to 40% of initial level indicates clonogenic survival of approximately 1%. Therefore a cell type in which GSH content can be depleted to 40% or less with the maximally achievable concentration of 4-OOH-CP in patients (20–25 µM) (Sladek *et al.*, 1984) may be considered 'sensitive'.

One of the important features of this assay is the ease and reliability with which a complete dose–response curve can be obtained for each tumour type (Figure 5). It is envisaged that the dose–response data obtained may ultimately aid in deter-

**Figure 3** a The effects of 4-OOH-CP treatment on cellular GSH content in the MLS human ovarian cancer cell line. Data shown are the average of three repeat experiments. b The concentration-cytotoxicity relationship of 4-OOH-CP for the MLS cell line. Results are from three independent experiments. All data points are means ±1 s.d.

mining the dose of cyclophosphamide needed to achieve a desired tumour response. This feature of the assay is of particular value because cyclophosphamide, with appropriate autologous bone marrow support, may be administered up to 15-fold above the conventional dosage (Smith *et al.*, 1983). With these escalated doses of cyclophosphamide, tumour response may become evident even when none was apparent at conventional dosages (Frei & Canellos, 1980; Antman *et al.*, 1987). Other important advantages of this GSH-targeted assay for clinical application include the following. (1) Cytotoxicity level greater than 1% clonogenic cell survival can be predicted. (2) Test results will be available within the same day. (3) Single cell suspensions are not a necessary requirement. (4) Micro-organism contamination, a frequently encountered difficulty in most predictive assays, is not a problem because of the short duration of the assay. (5) It is not necessary to establish a clonogenic cell survival assay which may prove problematic with human tumour samples.

The effectiveness of the GSH-targeted assay in predicting tumour cell sensitivity to 4-OOH-CP is founded on the special interaction between the active metabolites of cyclophosphamide and GSH (Hohorst *et al.*, 1976; Kwon *et al.*,

**Figure 4** The correlation between the extent of GSH depletion and clonogenic cell survival. a Human ovarian tumour cell lines. b Human lung and squamous carcinoma cell lines. All data are presented.

**Figure 5** a Clonogenic cells survival of three human tumour cell lines of diverse chemosensitivity to 4-OOH-CP. Cells were exposed to 4-OOH-CP for 3 h as described in Materials and methods. b The effects of 4-OOH-CP treatment for 3 h on the cellular GSH content of the same three cell lines.

1987). Evidence has been obtained demonstrating that the amount of the ultimate active metabolites, phosphoramide mustard (PM) and acrolein (AC), formed intracellularly from the spontaneous breakdown of 4-hydroxycyclophosphamide (4-OH-CP), is dependent on the extent of interaction between GSH and the toxic metabolites. GSH, by reacting with the intermediate metabolite aldophosphamide (AP), stabilises it from spontaneous breakdown to PM, thus minimising its cytotoxic effects. In a sensitive cell type, the GSH stabilising step is inadequate, leading to increased formation of AC and PM. The former can in turn further deplete cellular GSH. Consequently, a catastrophic cycle of GSH depletion, AP destabilisation and toxic metabolites formation is rapidly put into effect. We believe it is this mechanism that has rendered the cellular GSH status as such a sensitive indicator of tumour sensitivity to 4-OOH-CP.

Finally, a definite correlation between the steady-state cellular GSH content of tumour cell lines *in vitro* and their chemosensitivities to 4-OOH-CP (Figure 2) was also demonstrated. The considerable scatter in the data, however, does not allow the use of steady-state GSH content *per se* to precisely predict for sensitivity to 4-OOH-CP. Nevertheless, these data provide strong evidence that GSH plays a critical role in determining the success or failure of chemotherapy with cyclophosphamide.

We wish to thank Dr B. Fenton for helpful comments; and Amy Beikirch and Michele Chapman for excellent technical assistance.

### References

ADAMS, D.J., CARMICHAEL, J. & WOLF, C.R. (1986). Altered mouse bone marrow glutathione and glutathione transferase levels in response to cytotoxins. *Cancer Res.*, **45**, 1669.

ANTMAN, K., EDER, J.P., FREI, E., III (1987). High-dose chemotherapy with bone marrow support for solid tumors. *Important Adv. Oncol.*, **221**, 1987.

CARMICHAEL, J., FRIEDMAN, N., TOCHNER, Z. & 4 others (1986). Inhibition of the protective effect of cyclophosphamide by pretreatment with buthionine sulfoximine. *Int. J. Radiat. Oncol. Bio. Phys.*, **12**, 1191.

CROOK, T.R., SOUHAMI, R.L., WHYMAN, G.D. & MCLEAN, A.E.M. (1986). Glutathione depletion as a determinant of sensitivity of human leukemia cells to cyclophosphamide. *Cancer Res.*, **46**, 5035.

DRAEGER, U., PETER, G. & HOHORST, H.-J. (1976). Deactivation of cyclophosphamide (NSC-26271) metabolites by sulfhydryl compounds. *Cancer Treat. Rep.*, **60**, 335.

FREI, E. & CANELLOS, G.P. (1980). Dose: a critical factor in cancer chemotherapy. *Am. J. Med.*, **69**, 585.

GIARD, D.N., AARONSON, S.A., TODARO, J.G. & 4 others (1973). *In vitro* cultivation of human tumors: establishment of cell lines from a series of solid tumors. *J. Natl Cancer Inst.*, **51**, 1417.

GURTOO, H.L., HIPKENS, J.H. & SHARMA, S.D. (1981). Role of glutathione in the metabolism-dependent toxicity and chemotherapy of cyclophosphamide. *Cancer Res.*, **41**, 3584.

HILTON, J. (1984) Deoxyribonucleic acid crosslinking by 4-hydroxycyclophosphamide in cyclophosphamide-sensitive and -resistant L1210 cells. *Biochem. Pharmacol.*, **33**, 1867.

HOHORST, H.-J., DRAEGER, U., PETER, G. & VOELCKER, G. (1976). The problem of oncostatic specificity of cyclophosphamide (NSC-26271): studies on reactions that control the alkylating and cytotoxic activity. *Cancer Treat. Rep.*, **60**, 309.

KALLMAN, R.F., SILINI, G. & VAN PUTTEN, L.M. (1967). Factors influencing the quantitative estimation of the *in vivo* survival of cells from solid tumors. *J. Natl Cancer Inst.*, **39**, 539.

KWON, C.-H., BORCH, R.F., ENGEL, J. & NIEMEYER, U. (1987). Activation mechanisms of mafosfamide and the role of thiols in cyclophosphamide metabolism. *J. Medicinal Chem.*, **30**, 395.

LEE, F.Y.F. (1990). Glutathione diminishes the antitumor activity of 4-hydroperoxycyclophosphamide by stabilizing its spontaneous breakdown to alkylating metabolites. *Br. J. Cancer* (in press).

LEE, F.Y.F., SIEMANN, D., ALLALUNIS-TURNER, J. & KENG, P. (1988). Glutathione contents in human and rodent tumor cells in various phases of the cell cycle. *Cancer Res.*, **48**, 3661.

LEE, F.Y.F., SIEMANN, D.W. & SUTHERLAND, R.M. (1989). Changes in cellular glutatione content during adriamycin treatment in human ovarian cancer – a possible indicator of chemosensitivity. *Br. J. Cancer*, **60**, 291.

SIEMANN, D.W., FLAHERTY, A.A. & PENNEY, D.P. (1989). Effect of thiol manipulation on chemopotentiation by nitroimidazoles. *Int. J. Radiat. Oncol. Biol. Phys.*, **16**, 1341.

SLADEK, N.E. (1987). Oxazaphosphorines. In: *Metabolism and Action of Anti-Cancer Drugs*. Powis, G. & Prough, R.A. (eds) pp. 48–90. Taylor and Francis: London.

SLADEK, N.E., DOEDEN, D., POWERS, J.F. & KRIVIT, W. (1984). Plasma concentrations of 4-hydroxycyclophosphamide and phosphoramide mustard in patients repeatedly given high doses of cyclophosphamide in preparation for bone marrow transplantation. *Cancer Treat. Rep.*, **68**, 1247.

SMITH, I., EVANS, B.M., HARLAND, S. & MILLAR, J. (1983). Autologous bone marrow rescue is unnecessary after very high-dose cyclophosphamide. *Lancet*, **i**, 76.

SYKES, J.A., WHITESCARVER, J., JERNSTROM, P., NOLAN, J.F. & BYATT, P. (1970). Some properties of a new epithelial cell line of human origin. *J. Natl Cancer Inst.*, **45**, 107.

TOOLAN, H.W. (1954). Transplantable human neoplasms maintained iun cortisone-treated laboratory animals H.S#1; H.Ep#1; H.Ep#2; H.Ep#3; and H.Emb.Rb#1. *Cancer Res.*, **14**, 660.

WHILLANS, D.W. & RAUTH, A.M. (1980). An experimental and analytical study of oxygen depletion in stirred cell suspensions. *Radiat. Res.*, **84**, 97.


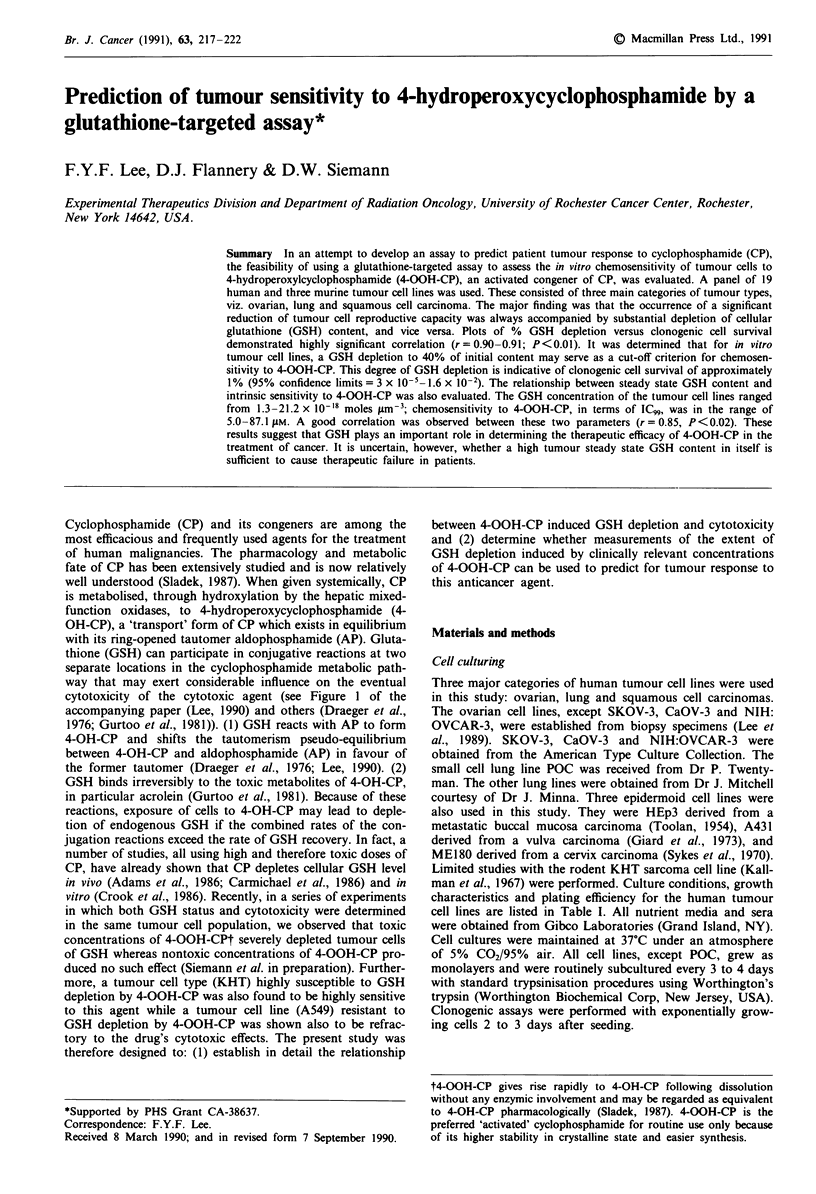

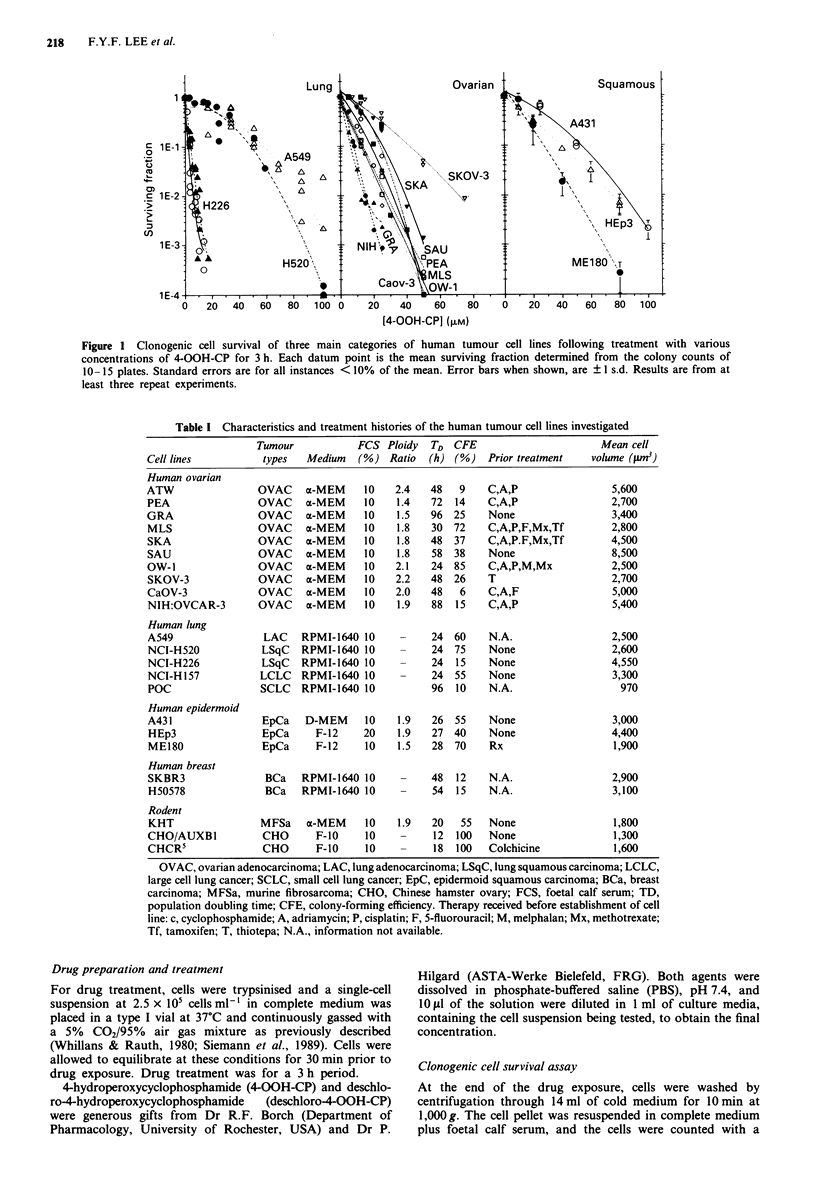

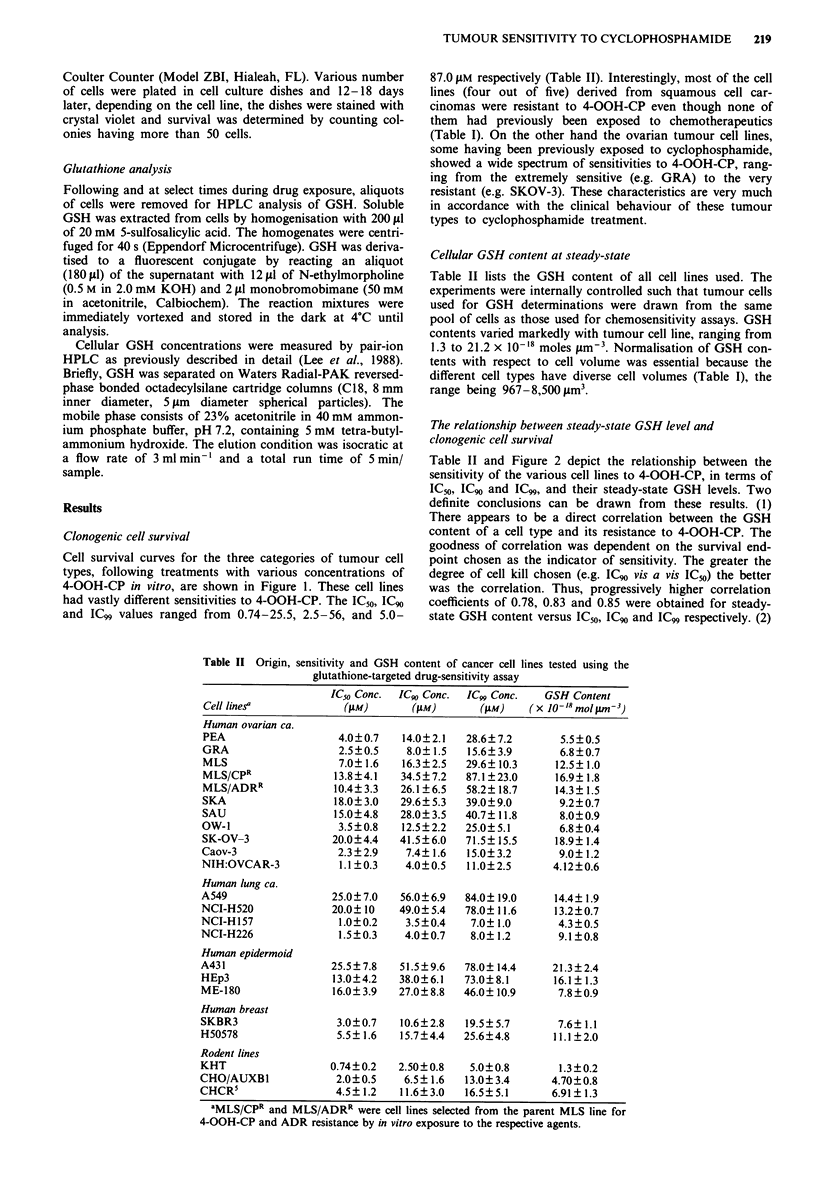

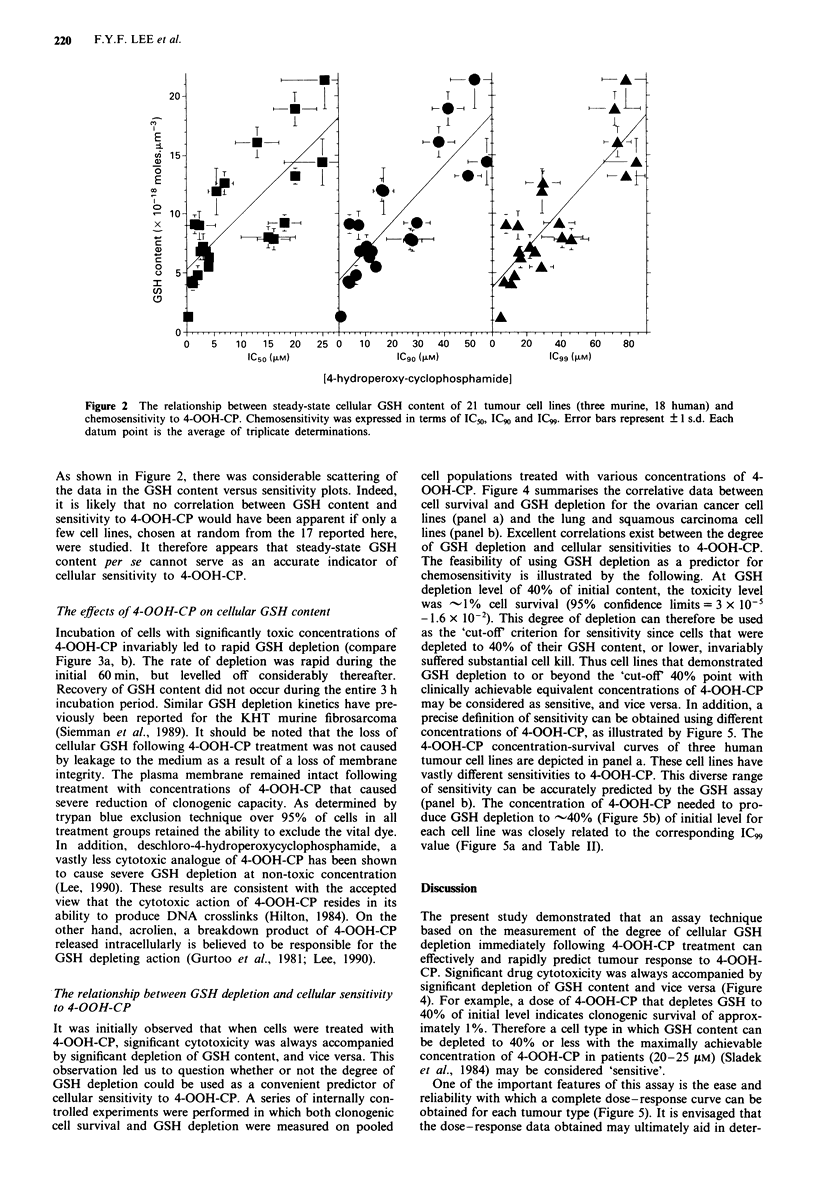

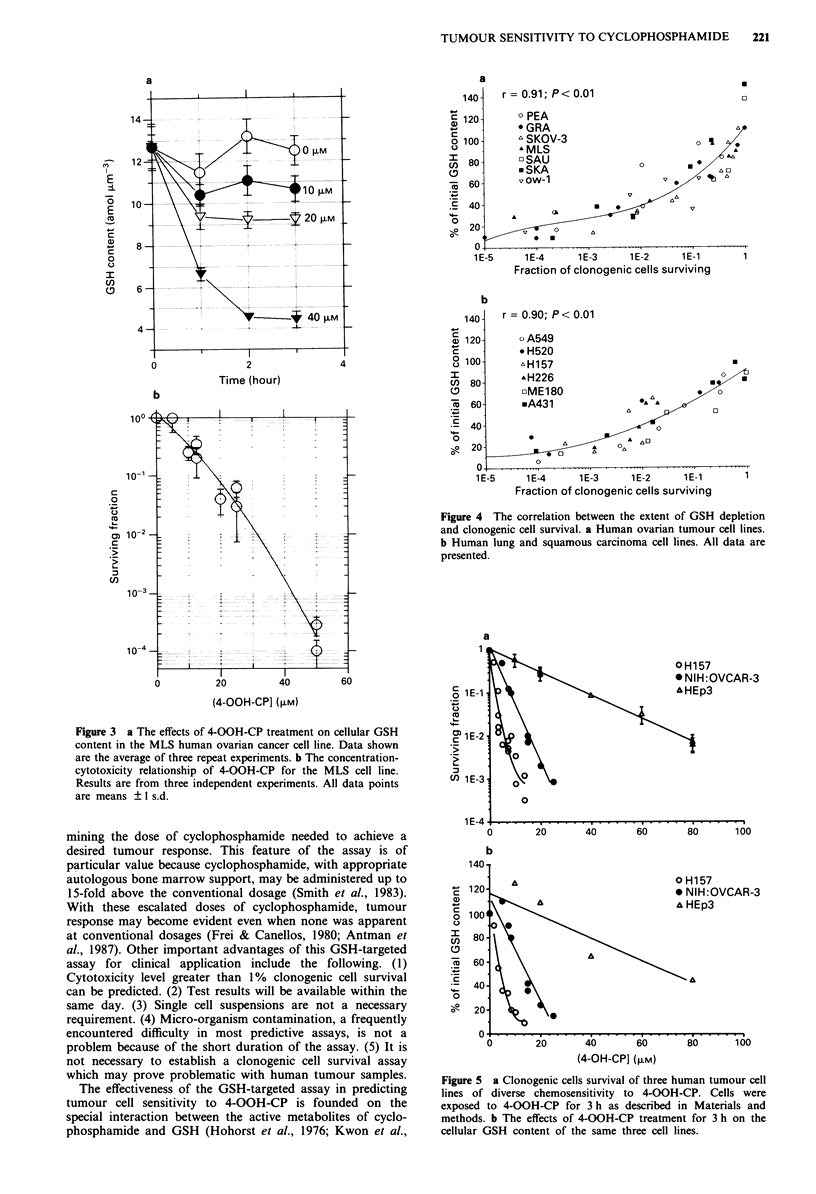

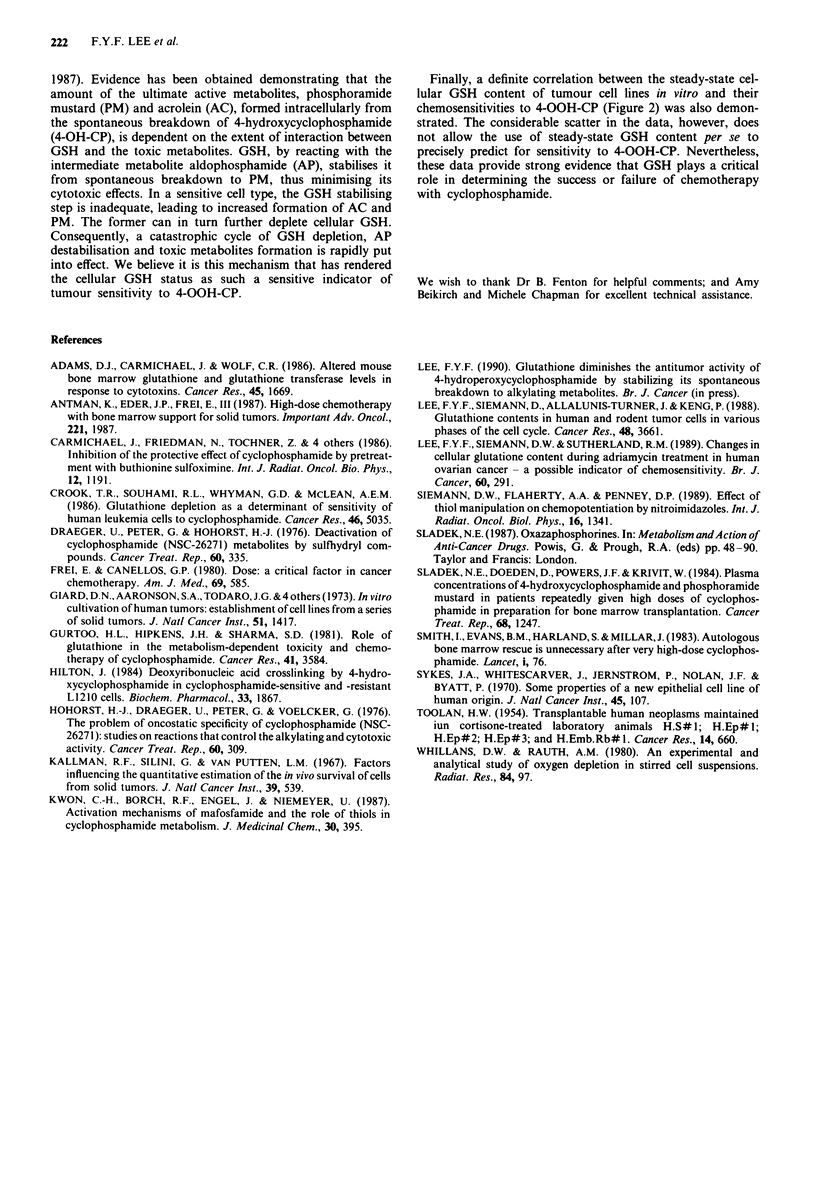


## References

[OCR_00749] Adams K. J., Carmichael J., Wolf C. R. (1985). Altered mouse bone marrow glutathione and glutathione transferase levels in response to cytotoxins.. Cancer Res.

[OCR_00759] Carmichael J., Friedman N., Tochner Z., Adams D. J., Wolf C. R., Mitchell J. B., Russo A. (1986). Inhibition of the protective effect of cyclophosphamide by pre-treatment with buthionine sulfoximine.. Int J Radiat Oncol Biol Phys.

[OCR_00765] Crook T. R., Souhami R. L., Whyman G. D., McLean A. E. (1986). Glutathione depletion as a determinant of sensitivity of human leukemia cells to cyclophosphamide.. Cancer Res.

[OCR_00774] Frei E., Canellos G. P. (1980). Dose: a critical factor in cancer chemotherapy.. Am J Med.

[OCR_00778] Giard D. J., Aaronson S. A., Todaro G. J., Arnstein P., Kersey J. H., Dosik H., Parks W. P. (1973). In vitro cultivation of human tumors: establishment of cell lines derived from a series of solid tumors.. J Natl Cancer Inst.

[OCR_00783] Gurtoo H. L., Hipkens J. H., Sharma S. D. (1981). Role of glutathione in the metabolism-dependent toxicity and chemotherapy of cyclophosphamide.. Cancer Res.

[OCR_00788] Hilton J. (1984). Deoxyribonucleic acid crosslinking by 4-hydroperoxycyclophosphamide in cyclophosphamide-sensitive and -resistant L1210 cells.. Biochem Pharmacol.

[OCR_00793] Hohorst H. J., Draeger U., Peter G., Voelcker G. (1976). The problem of oncostatic specificity of cyclophosphamide (NSC-26271): Studies on reactions that control the alkylating and cytotoxic activity.. Cancer Treat Rep.

[OCR_00799] Kallman R. F., Silini G., Van Putten L. M. (1967). Factors influencing the quantitative estimation of the in vivo survival of cells from solid tumors.. J Natl Cancer Inst.

[OCR_00804] Kwon C. H., Borch R. F., Engel J., Niemeyer U. (1987). Activation mechanisms of mafosfamide and the role of thiols in cyclophosphamide metabolism.. J Med Chem.

[OCR_00814] Lee F. Y., Siemann D. W., Allalunis-Turner M. J., Keng P. C. (1988). Glutathione contents in human and rodent tumor cells in various phases of the cell cycle.. Cancer Res.

[OCR_00819] Lee F. Y., Siemann D. W., Sutherland R. M. (1989). Changes in cellular glutathione content during adriamycin treatment in human ovarian cancer--a possible indicator of chemosensitivity.. Br J Cancer.

[OCR_00825] Siemann D. W., Flaherty A. A., Penney D. P. (1989). Effect of thiol manipulation on chemopotentiation by nitroimidazoles.. Int J Radiat Oncol Biol Phys.

[OCR_00835] Sladek N. E., Doeden D., Powers J. F., Krivit W. (1984). Plasma concentrations of 4-hydroxycyclophosphamide and phosphoramide mustard in patients repeatedly given high doses of cyclophosphamide in preparation for bone marrow transplantation.. Cancer Treat Rep.

[OCR_00842] Smith I. E., Evans B. D., Harland S. J., Millar J. L. (1983). Autologous bone marrow rescue is unnecessary after very-high-dose cyclophosphamide.. Lancet.

[OCR_00847] Sykes J. A., Whitescarver J., Jernstrom P., Nolan J. F., Byatt P. (1970). Some properties of a new epithelial cell line of human origin.. J Natl Cancer Inst.

[OCR_00852] TOOLAN H. W. (1954). Transplantable human neoplasms maintained in cortisone-treated laboratory animals: H.S. No. 1; H.Ep. No. 1; H.Ep. No. 2; H.Ep. No. 3; and H.Emb.Rh. No. 1.. Cancer Res.

[OCR_00857] Whillans D. W., Rauth A. M. (1980). An experimental and analytical study of oxygen depletion in stirred cell suspensions.. Radiat Res.

